# Genome Wide Association Studies in Multiple Spinach Breeding Populations Refine Downy Mildew Race 13 Resistance Genes

**DOI:** 10.3389/fpls.2020.563187

**Published:** 2020-10-21

**Authors:** Gehendra Bhattarai, Ainong Shi, Chunda Feng, Braham Dhillon, Beiquan Mou, James C. Correll

**Affiliations:** ^1^Department of Horticulture, University of Arkansas, Fayetteville, AR, United States; ^2^Department of Plant Pathology, University of Arkansas, Fayetteville, AR, United States; ^3^Department of Plant Pathology, Fort Lauderdale Research and Education Center, University of Florida, Davie, FL, United States; ^4^Crop Improvement and Protection Research Unit, United States Department of Agriculture, Agricultural Research Service, Salinas, CA, United States

**Keywords:** spinach, downy mildew, oomycete, disease resistance, mapping, GWAS, candidate gene

## Abstract

Downy mildew, caused by the oomycete *Peronospora effusa*, is the most economically important disease on spinach. Fourteen new races of *P. effusa* have been identified in the last three decades. The frequent emergence of new races of *P. effusa* continually overcome the genetic resistance to the pathogen. The objectives of this research were to more clearly map the downy mildew resistance locus *RPF*1 in spinach, to identify single nucleotide polymorphism (SNP) markers associated with the resistance, and to refine the candidate genes responsible for the resistance. Progeny from populations generated from crosses of cultivars resistant (due to *RPF*1) to race 13 of *P. effusa* (Swan, T-Bird, Squirrel, and Tonga) with race 13 susceptible cultivars (Whale and Polka) were inoculated and the downy mildew disease response determined. Association analysis was performed in TASSEL, GAPIT, PLINK, and GENESIS programs using SNP markers identified from genotyping by sequencing (GBS). Association analysis mapped the race 13 resistance loci (*RPF*1) to positions 0.39, 0.69, 0.94-0.98, and 1.2 Mb of chromosome 3. The associated SNPs were within 1–7 kb of the disease resistance genes Spo12784, Spo12719, Spo12905, and Spo12821, and 11–18 Kb from Spo12903. This study extended our understanding of the genetic basis of downy mildew resistance in spinach and provided the most promising candidate genes Spo12784 and Spo12903 near the *RPF*1 locus, to pursue functional validation. The SNP markers may be used to select for the resistant lines to improve genetic resistance against the downy mildew pathogen and in developing durably resistant cultivars.

## Introduction

Spinach (*Spinacia oleracea*) is an important cool-season leafy vegetable crop. Spinach is dioecious, wind-pollinated, and a highly heterozygous species. The United States is the second-largest producer of spinach after China. The United States annually produces approximately 475,000 tons of spinach with a product value of 425 million dollars (USDA-NASS, 2019). Most of the fresh market spinach in the US is produced during mildly-cool seasons in the valleys of California and Arizona (Koike et al., 2011). Spinach is a diploid crop with six chromosomes (2*n* = 2*x* = 12). Spinach is nutritious and an excellent source of health-promoting compounds and nutrients (Morelock and Correll, 2008). There is an increasing demand for spinach in the United States and organic production comprises around 50% of the total production.

Downy mildew, caused by the obligate oomycete *Peronospora effusa* [*P. farinosa* f. sp. *spinaciae* (*Pfs*)], is the most economically important disease of spinach. Seventeen unique races of *P. effusa* have been documented (Satou et al., 2006; Irish et al., 2007; Feng et al., 2014, 2018b) in spinach, of which fourteen were reported in the last three decades. New races of *P. effusa* have emerged which have overcome newly deployed genetic resistance making downy mildew a major challenge for the sustainability of the spinach industry. A recent report shows the degree of genetic variation within the spinach downy mildew pathogen population (Lyon et al., 2016). The presence of opposite mating types in the population of *P. effusa* was demonstrated among isolates in California based on controlled crosses (Dhillon et al., 2020). Downy mildew can be controlled using fungicides, to a limited degree, biofungicides, and crop rotations. However, chemical control is costly and limited to conventional spinach production (Correll et al., 2011; Klosterman, 2016; Subbarao et al., 2018). The utilization of genetic resistance in developing new resistant cultivars is the most promising disease management approach in crops, and particularly in organic production, where the use of resistant cultivars is the only viable disease management option.

Most of the downy mildew resistant spinach cultivars were bred using single-gene resistance against the various races of *P. effusa.* Six different *RPF* loci (Resistance to *Peronospora farinosa*) were hypothesized to provide resistance to races of *P. effusa* (Correll et al., 2011), and *RPF* has been genetically characterized (Feng et al., 2018a). The *RPF*1 locus, governed by a single dominant allele, was mapped to chromosome 3, and a codominant marker DM1 was 1.7 cM from the *RPF*1 locus (Irish et al., 2008). The *P. effusa* resistance loci *RPF*1, *RPF*2, and *RPF*3 were mapped to a 1.5 Mb region of chromosome 3, and PCR markers that can distinguish the *RPF* loci were reported (Feng et al., 2015, 2018a). The *RPF*1 locus was further narrowed to a 0.89 Mb region between 0.34 and 1.23 Mb, and the three most likely candidate genes were predicted following protein homology comparison between the resistant and susceptible lines (She et al., 2018). Five *P. effusa* resistance candidate genes were postulated based on the NBS-LRR domains in the spinach genome sequence (Xu et al., 2017).

The rapid emergence of new races that are overcoming the resistance genes in commercial cultivars has increased research attention toward downy mildew disease management in spinach breeding programs (Morelock and Correll, 2008). Identifying additional resistance sources against the known races of *P. effusa* and expanded understanding of the mechanism of genetic resistance could provide an increase in genetic resources to improve the durability of resistance. However, a challenge remains in the gaps in our knowledge of genetics and molecular aspects of qualitative and quantitative host resistance, and pathogen virulence factors and the mechanism of pathogen evolution. Genetic characterization of the resistance sources and identification of gene-based markers will facilitate R-gene pyramiding. Furthermore, identification of susceptibility genes (*S*-genes) may allow an alternative and promising strategy to develop resistant cultivars by loss-of-function of the S-genes to achieve durable and non-race-specific resistance (Bai et al., 2008; Pavan et al., 2011; Pessina et al., 2016; Wang et al., 2016).

Genetic linkage mapping and genome wide association studies (GWAS) have long been used to identify linked markers or associated genomic regions controlling the phenotypic expression. Spinach cultivars Swan, Squirrel, Tonga, and T-Bird were resistant to race 13 of *P. effusa*. Populations developed from crosses of these resistant cultivars with other susceptible cultivars were screened for resistance against race 13 of *P. effusa* to map the resistance loci using the association mapping approach. Biparental QTL mapping requires the development of progeny segregating for a trait of interest, while GWAS allows mapping the trait in diverse germplasm or a mixed population. Many traits in plants and animals have been mapped using the GWAS approach (Wallace et al., 2016; Guo et al., 2017; Minamikawa et al., 2018).

This study used the GWAS method to identify genomic regions controlling resistance to the downy mildew pathogen from multiple biparental populations. The specific objective of this study was to fine map the race 13 resistance loci from multiple segregating populations to identify the SNP markers associated with the resistance. Associated genomic regions were used to identify and refine the candidate genes involved in providing resistance to the downy mildew pathogen.

## Materials and Methods

### Plant Materials and Populations

**FIGURE 1:**
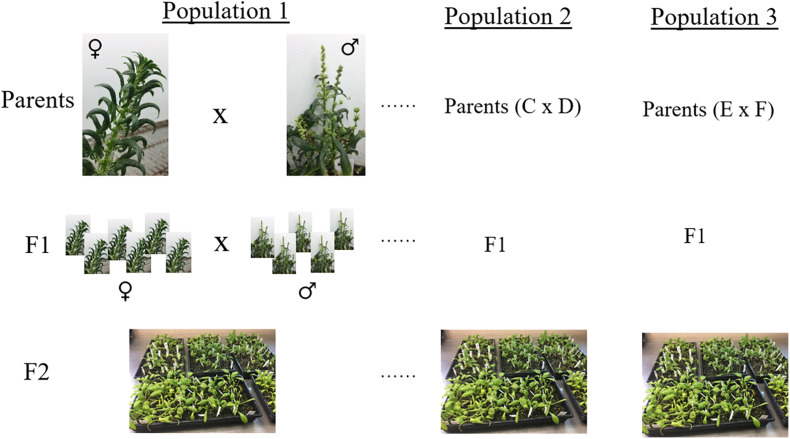
Schematic representation of the breeding population generated in this study. Spinach inbred lines resistant to race 13 of *P. effusa* were crossed with a susceptible inbred to produce F1 seeds. The heterozygous resistant F1 cultivar was grown with susceptible F1 cultivar, allowed to inter-cross in a crossing block, and F2 seeds were generated. Multiple segregating populations were phenotyped and genotyped that constitute the association panel in this study.

Breeding populations segregating for resistance to race 13 of *P. effusa* (isolate UA0510C) from crosses of resistant cultivars Swan, Squirrel, Tonga, and T-Bird (Table 1) with susceptible cultivars Whale and Polka were evaluated. All race 13 resistant cultivars contain the *RPF*1 locus as all other known *RPF*2 through *RPF*6 loci are susceptible to race 13, while the susceptible cultivar Whale and Polka contains *RPF*3 and *RPF*3/*RPF*5 locus (Feng et al., 2014, 2018a,b). Seeds of F1 cultivars were obtained from seed companies (Swan, Squirrel, Tonga, T-Bird, Whale, and Polka). The initial F1 seeds obtained from commercial seed companies were generated by crossing resistant and susceptible inbred lines for a particular *RPF* locus, and the F1 seeds contain a single *RPF* allele. The F1 seeds of each cultivar were grown in the greenhouse for 3 weeks at the USDA experiment station in Salinas, CA, United States. Around 32-68 F1 plants from each cross were moved to a single isolator block, allowed to intercross, and F2 seed were generated. Seed harvested from each of the female plants represent a segregating progeny population from the respective parental cultivars and seeds were bulked from each isolator (Figure 1). Initially, a small set of 25–50 plants of each F2 populations and parent cultivars were screened for downy mildew disease segregation following race 13 inoculation at the Rosen Alternative Pest Control Center, University of Arkansas. Based on pre-evaluation, the F2 populations were selected and tested for resistance against race 13 of *P. effusa*. Multiple populations were pooled that constitute the association panel used in this study (Table 1). Parental cultivars and the differentials, including NIL1 and Viroflay, were included in the disease screening as controls. Seeds were sown in 25-cm × 50-cm plastic trays filled with potting soil (Sun Gro Horticulture, Canada). Each plant tray contained ten rows, and 10–15 seed per row were planted. After germination, 6–8 plants were kept per row and were labeled using a plant tag. Plants were grown in the greenhouse (25°C) for 2 weeks, watered daily, and fertilized weekly using Miracle-Gro^®^ All Purpose Plant Food.

**TABLE 1:** Spinach progeny populationsegregating for downy mildew response following inoculation with race 13 of *P. effusa.*

Parents	Resistant	Susceptible	Total	χ^2^	*P*
Resistance from Swan					
Whale × Swan	58	45	103	1.64	0.20
GBS^a^	34	34	68		
Polka × Swan	40	38	78	0.05	0.82
GBS	4	9	13		
Resistance from Squirrel					
Squirrel × Polka	23	41	64	5.06	0.02
GBS	8	10	18		
Whale × Squirrel	19	43	62	9.29	0.00
GBS	18	12	30		
Resistance from Tonga					
Polka × Tonga	28	64	92	14.09	0.00
GBS	10	9	19		
Resistance from T-Bird					
Polka × T-Bird	17	57	74	21.62	0.00
GBS	14	7	21		
GBS additional sources					
Parents and controls	6	7	13		
Other unknowns	–	–	8		
Total Phenotyped	185	288	473		
Total (GBS)^b^	94	88	190		
Filtering					
Other unknowns			8		
Individual missing			6		
High heterozygosity			2		
Total (Association panel)^c^	89	85	174		
Association panel	32	33	65		
(Whale × Swan)^d^					

*Cultivar Swan, Squirrel, Tonga, and T-Bird are resistant to race 13 of *P. effusa*, and Polka and Whale are susceptible cultivars. Chi-square goodness of fit test was for a 1:1 ratio. ^*a*^Number of progeny lines pursued to genotype using the genotyping by sequencing (GBS) approach (Elshire et al., 2011). ^*b*^Total number of lines pursued to genotype. ^*c*^Total number of lines pursued to conduct association analysis that remained after filtering for lines that were not phenotyped the same time with all other panels, high missing SNP data, and high heterozygosity. ^*d*^Progeny lines from a cross of Whale and Swan were only used to perform independent association analysis to identify resistant loci specific to cultivar Swan.*

### Downy Mildew Inoculation and Disease Screening

Before inoculation, one leaf from each of the labeled seedlings was excised and stored for DNA extraction. Seedlings in trays were inoculated following the standard inoculation method (Feng et al., 2014, 2018b). Briefly, the inoculation assay involves growing plants for 2 weeks in the greenhouse. Fresh inoculums were prepared every week on susceptible cultivar Viroflay and conidia were washed off from the infected leaves in cold (4°C) distilled water. The spore suspension diluted to 10^5^ spores per ml was used to spray inoculate using a Badger basic spray gun (model 250) until the leaves were wet. Inoculated plants in trays were incubated in a dew chamber (18°C) for 24 h in dark, moved to a growth chamber (18°C, 12 h dark-light cycle) for 5 days, and finally returned to the dew chamber (18°C) for 24 h to induce sporulation. The disease reactions of each plant were rated 7 days post inoculation (dpi) for the presence and absence of sporulation on cotyledons and true leaves. A plant was scored as “resistant” if both cotyledons and leaves show no sporulation, otherwise scored as “susceptible.” Each line was phenotyped for downy mildew based on disease reaction of a single plant and by following a one-time inoculation, and as such, to minimize phenotyping error, a set of high confident resistant plants were selected for genotyping and genetic analysis. An immune plant in the vicinity of diseased plants was pursued to genotype, while the presence of many resistant plants in a single tray-row was not included for genotyping.

### Sequencing and Marker Discovery

The seedling populations were sequenced following the GBS method (Elshire et al., 2011). Genomic DNA was extracted from leaves stored at −80°C using the CTAB (cetyl trimethylammonium bromide) method. Genomic DNA was checked for quality on 1% agarose gel, quantified in a NanoDrop, and DNA meeting the requirements were submitted for sequencing at the UW-Madison Biotech center.

DNA quality and integrity were re-evaluated at the Biotech labs using Quant-IT PicoGreen fluorescent dye (Thermo Fisher, Waltham, MA, United States). Each sample DNA was digested using *Ape*KI restriction enzyme. Digested DNA fragments were ligated with unique barcode adapters and Illumina adapters and samples were pooled in equal proportion to construct GBS libraries, as described in Elshire et al. (2011). Finally, the GBS libraries were amplified, purified, and sequenced as 150 bp paired-end reads on the Illumina NovaSeq machine (Illumina, San Diego, CA, United States).

Raw reads were preprocessed to remove sequencing adapters and to filter for low-quality bases for a minimum quality of Q20 using skewer program (Jiang et al., 2014). Remaining good quality reads were demultiplexed and aligned to the six chromosomal scaffolds of the spinach reference genome^1^ (Xu et al., 2017) using Bowtie 2 software (Langmead and Salzberg, 2012). The aligned sequences were analyzed in the TASSEL GBS version 2 pipeline (Bradbury et al., 2007; Glaubitz et al., 2014) for genotyping and Single Nucleotide Polymorphism (SNP) calling. Multiallelic SNPs were filtered using VCFtools v0.1.15 (Danecek et al., 2011) to keep only biallelic SNPs. For quality control, SNPs were filtered in PLINK v1.9 (Purcell et al., 2007; Chang et al., 2015). SNP with missing data >20%, plants with >20% missing data, minor allele frequency (MAF < 0.05), and plants with heterozygosity rate deviating by more than three standard deviations from the mean were removed. Final filtered SNP distribution on six spinach chromosomes was drawn using CMplot package in R.

### Population Structure and Clustering

Using a model-based clustering algorithm in ADMIXTURE v1.22 (Alexander et al., 2009), the subpopulation structure of the spinach panels was analyzed and individual lines were assigned to specific sub-population groups. The filtered SNP dataset was pruned for linkage disequilibrium (LD) in PLINK (–indep-pairwise 50 5 0.1 option) to remove correlated pairs of SNPs. LD pruned SNPs were used to run ADMIXTURE analysis with ten-fold cross-validation for one to ten groups. An optimum number of subpopulation groups were determined based on the lowest cross-validation error, and the Q matrices were used to draw the barplot to visualize the clustering of spinach lines. ADMIXTURE estimates the probability of the membership of an individual to each of the clusters. A cutoff probability of 0.75 was used to assign an individual to a cluster, and individuals with less than 0.75 membership probabilities were assigned to an admixed group.

The principal component analysis (PCA) was run using the identity by state (IBS) matrix calculated with the LD pruned SNPs in PLINK. The number of principal components (PC) was chosen according to the optimum subpopulation determined in ADMIXTURE to use as a covariate in PLINK to control population structure, and a PCA plot was drawn using R package ggplot2.

### Genome Wide Association Analysis

Genome wide association studies was performed on the phenotype and genotyped spinach population in two sets; the first set consisted of the complete sets of genotyped panels, while the second set consisted of population segregating only from cultivar Swan and Whale. The resistant and susceptible plants scored as 0 and 1 were used as the phenotype data set.

The first GWAS analysis was performed using the mixed linear model (MLM) by including PCA and kinship matrices in TASSEL 5.2.31 (Bradbury et al., 2007). A second association analysis was performed using the enriched compressed mixed linear model (EcMLM) in GAPIT (Lipka et al., 2012). A third association analysis was run using the logistic regression model in PLINK v1.9, which has been widely used in case-control association studies. Association analysis was performed in PLINK using the PCA clusters generated on the matrix of IBS sharing of all individual pairs, and the PCA matrices were used as a covariate to control for genetic relatedness. A final GWAS was run using the logistic mixed model (LMM) in the GENESIS R Bioconductor package. GENESIS uses mixed models to test genetic associations where PC-AiR computed principal components are used as fixed effect covariates to account for unknown and known relatedness of the lines (Conomos et al., 2015). A kinship matrix (or genetic relationship matrix) was estimated from PC-Relate to use as a random effect to account for phenotype correlation due to the genetic similarity among the lines (Conomos et al., 2016). The principal component and kinship matrix were calculated in GENESIS using LD pruned SNPs. The Kinship matrices were added as a random effect in the null model, and the principal components were included as fixed-effects covariates in the GWAS model.

Association analysis was performed for the second time using the progeny population obtained from a cross of Whale and Swan using the models as described for the first set. Manhattan plots and QQplots for all association models were drawn using qqman and CMplot package in R. Bonferroni significance threshold (0.05/*n*) was used and −log_10_(*P*) > 5.3 were reported for the first set of association panel and −log_10_(*P*) > 5.2 for the second set.

### Haplotype Analysis

Haplotype block analysis was performed within the associated region obtained from the whole panel in the Haploview 4.2 (Barrett et al., 2005). The haplotype blocks were defined using the confidence interval method of Gabriel et al. (2002), and a pairwise measure of LD (*r*^2^) was plotted as a heatmap. Haplotype analysis was further performed in the Plink v1.07 (Purcell et al., 2007) using default settings. The haplotype alleles from the haplotype blocks were utilized as multiallelic markers to conduct haplotype-based association analysis using the logistic regression and linear regression models in Plink v1.07.

### Candidate Gene Identification

High confident SNPs identified from multiple association models analyzed using two association panels were used to search for candidate genes in the spinach genome database^1^ (Collins et al., 2019). Genes 5, 10, and 20 Kb near the peak associated SNPs were examined for annotated functions, and all genes predicted to provide disease resistance against plant pathogens were considered as potential candidate genes, and their predicted functions were reported. From the haplotype analysis, genes within and near the haplotype blocks that include the associated SNPs were searched to refine the region and to increase the confidence of the identified candidate genes.

## Results

### Resistance Response to *P. effusa* in the Association Panel

Spinach cultivars differing in resistance response to races of *P. effusa* were purposefully crossed to generate segregating populations (Figure 1) to use in genetic characterization of resistance against the downy mildew pathogen. Spinach breeding populations segregating for resistance to race 13 of *P. effusa* from cultivars Swan, T-Bird, Squirrel, and Tonga from the initial pre-evaluation trial (data not shown) were later evaluated for resistance in the greenhouse and growth chamber facility following the standard inoculation protocol (Feng et al., 2014, 2018b) and used to investigate the genetic resistance in this study (Table 1). Inoculated plants were scored as resistant if there were no signs of the pathogen on cotyledons and leaves; otherwise, they were scored as susceptible. Resistant and susceptible control cultivars ‘NIL1’ and ‘Viroflay’ and the parent cultivars of the segregating population showed expected mildew response during all experiments. Progeny population segregating for race 13 resistance from cultivar Swan (Whale × Swan and Polka × Swan) showed a good fit to the expected 1:1 segregation ratio (Table 1). However, the progeny population segregating from Squirrel, Tonga, T-Bird, although expected to segregate 1:1, did not fit the expected segregation and showed an excess of susceptible plants. Of the total phenotyped panel, a subset of seedling lines from each population was selected for genotyping (Table 1). Association analysis was conducted using the binary disease score in a panel of 174 spinach lines (89 resistant and 85 susceptible lines) that remained after filtering for lines with unknown phenotype, high SNP missingness, and high heterozygosity (Table 1). Furthermore, the progeny population derived from a cross of race 13 resistant cultivar Swan and susceptible cultivar Whale, was used as an independent panel to run a second set of association analysis.

### Sequencing and SNP Discovery

**FIGURE 2:**
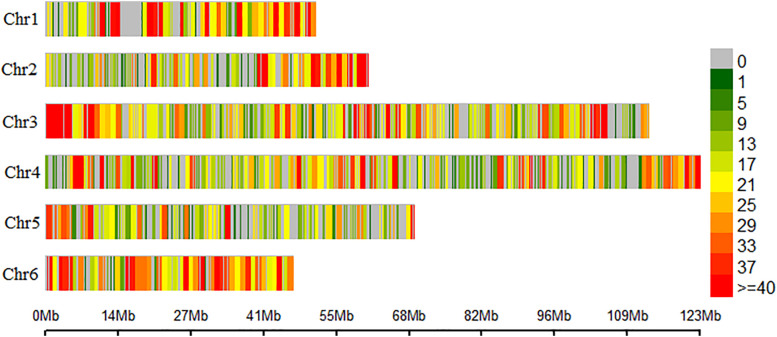
Distribution of the single nucleotide polymorphism (SNP) marker on the six chromosomes of spinach. Spinach chromosomes are on the vertical axis. Chromosome length in Mb is on the horizontal axis, and the color represents the number of SNPs per window, SNP density.

In total, 675 million raw reads were generated from the Illumina NovaSeq run with an average of 3.4 million reads/sample. After filtering for sequencing adapters and low-quality bases, 652 million (98%) good reads were aligned to six chromosomes of the spinach reference genome^1^ (Xu et al., 2017). In total, 165,747 SNPs were identified in the TASSELv2 pipeline. Filtering for INDELS (1182 variants removed) and keeping only biallelic SNPs (70,302 variants removed) retained 94,263 SNPs in VCFtools. Also, 182 lines remained after filtering for blanks and samples with unknown phenotypes. SNPs were further filtered in PLINK for missing SNP calls above 20% (48,660 variants removed), individuals with more than 20% missingness (6 plants removed), keeping minor allele frequency above 5% (31,555 variants removed) and HWE (>1e-07) removed 4,265 variants. Similarly, filtering for high heterozygosity removed two spinach lines making a final filtered dataset containing 9,783 SNPs and 174 spinach lines. The distribution of SNPs on the six chromosomes of spinach is presented as a density plot (Figure 2). The second association panel consisted of 65 spinach lines obtained from a cross of Swan and Whale and 7,633 SNPs that remained after filtering for low-quality SNPs.

### Population Structure and Principal Component Analysis

**FIGURE 3:**
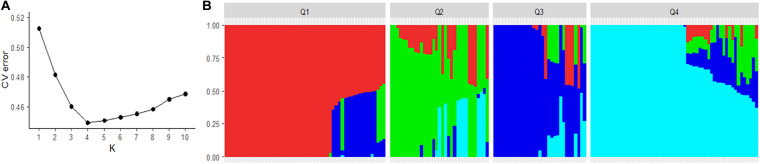
Population structure of the spinach panel generated from the ADMIXTURE run. **(A)** Optimum K was determined using the minimum cross-validation errors in the data for K. **(B)** Classification of spinach lines in the association panel into four genetic sub-population. The horizontal axis represents the spinach lines, and the vertical axis of the plot represents the probability of genotypes belonging to different genetic groups. Spinach lines membership proportion to each population group is indicated by a unique color, red (Q1), green (Q2), blue (Q3), and turquoise (Q4).

**FIGURE 4:**
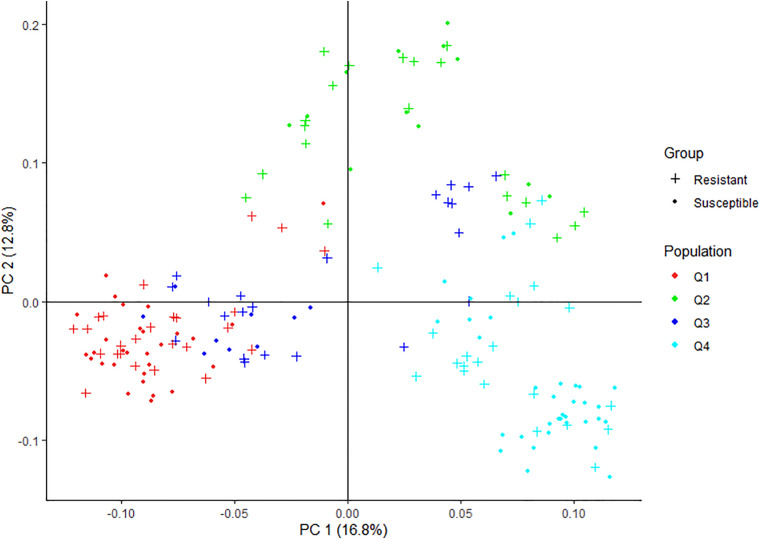
Graphical plot of the principal component analysis (PCA) of the spinach lines in the association panel. The horizontal and vertical axis are the first and second principal components (PC), and the variance explained by each PC is noted. Colors correspond to members of subpopulation Q1 (red), Q2 (green), blue (Q3), and turquoise (Q4). Resistant and susceptible genotypes are presented by “plus” and “filled circle” signs. The same colors were used for the subpopulations generated from ADMIXTURE and PCA plot in Figures 2, 3.

Pruning for high LD SNPs to remove correlated pairs of SNPs in PLINK retained 3634 SNPs. The LD pruned SNPs were used to analyze the genetic structure of the spinach panel on ADMIXTURE software. The ADMIXTURE cross-validation error supports four main clusters in the spinach panel (Figure 3A). A membership cutoff with maximum probability assignment was used to divide spinach genotypes into the four sub-population, and there were 54, 33, 31, 56 genotypes assigned to subpopulation Q1, Q2, Q3, and Q4, respectively (Figure 3B). The principal component analysis was performed in PLINK. The first two principal components accounting for 16.8 and 12.8% of the total genetic variation differentiate the genotypes into genetically distinct sub-groups of the association panel (Figure 4), and the resistant and susceptible genotypes were equally present in all the sub-populations. The first four dimensions of the PCA on the matrix of IBS sharing of all pairs of individuals were used as covariates in PLINK and in other mixed models to control population stratification. Next, for the population panel segregating from cultivar Swan and Whale, the first two principal components were used as covariates in the mixed linear models (Supplementary Figure 1).

### GWAS Analysis With Single SNP Markers

Association analysis was conducted using 9,783 GBS-generated SNPs on a panel of 174 spinach genotypes to identify the genetic loci governing the resistance to race 13 of *P. effusa*. GWAS models were run on multiple programs to determine consistent associations and to avoid spurious associations. A Bonferroni correction threshold (LOD value >5.3) was employed to detect the significance of the GWAS result. Significant SNPs detected on multiple programs and association models were of high-confidence and were considered to associate with resistance to race 13 of *P. effusa*. The high-confident SNPs identified from the association study were used to explore for the candidate genes.

**FIGURE 5:**
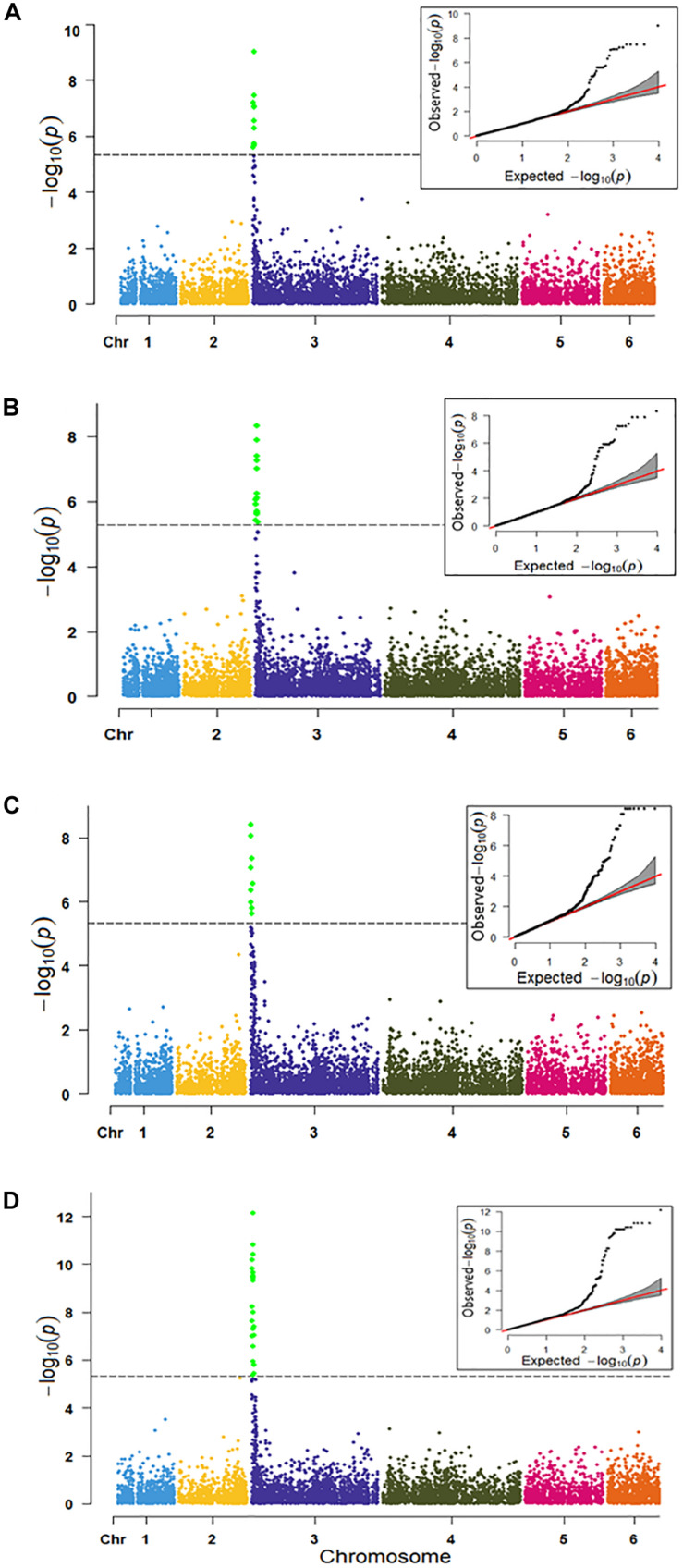
Manhattan and QQ-plots of genome wide associations of the race 13 of *P. effusa* resistance in spinach in TASSEL **(A)**, GAPIT **(B)**, PLINK **(C)**, and GENESIS **(D)** model. The horizontal and vertical axis represents the genomic position of the SNP and association power for each SNP with the trait expressed as –log_10_(*P*-value). The dashed line shows the Bonferroni-corrected genome wide threshold.

Association analysis was performed in TASSEL using the MLM model by including four principal components and kinship matrices to control population structure and family relatedness. Twenty-two significant SNPs were detected in the MLM model (Table 2 and Figure 5A). All significant SNPs were present in the 0.395–1.270 Mb region of chromosome 3. The phenotypic variance (*R*^2^) explained by the three SNPs loci averages 20% with the maximum variation of 27.3% for SNP S3_1210582. Similarly, the EcMLM model in GAPIT identified 25 significant SNPs (Table 3 and Figure 5B).

**TABLE 2:** SNP markers associated with resistance to race 13 of *P. effusa* in spinach breeding populations. Progeny population was generated from a cross of resistant cultivars Swan, squirrel, Tonga, T-Bird with susceptible cultivars Whale and Polka.

SNP marker^a^	Alleles^b^	MAF	-log_10_ *P*-value^c^				R^2^ (%) MLM^d^	Nearest genes^e^	Annotation	Kb away from the gene
			TASSEL	GAPIT	PLINK	GENESIS				
Association panel comprises seedlings from all genotyped population										
S3_325976	T:**C**	0.39	4.85	4.83	7.05	8.23	13.87	–	–	–
S3_325991	A:**C**	0.39	4.85	4.83	7.05	8.23	13.87	–	–	–
S3_326104	C:**T**	0.38	2.79	3.67	6.36	7.00	7.75	–	–	–
S3_395843	A:**C**	0.44	7.20	6.05	8.06	9.80	21.58	Spo12719	Receptor-like kinase	5.98 Kb downstream
S3_396000	C:**A**	0.45	5.59	5.92	8.41	10.18	16.20	Spo12719	Receptor-like kinase	6.13 Kb downstream
S3_396030	G:**A**	0.45	5.59	5.92	8.41	10.18	16.20	Spo12719	Receptor-like kinase	6.16 Kb downstream
S3_473572	**C**:T	0.19	4.60	5.42	5.98	7.62	13.12	–	–	–
S3_989568	**C**:T	0.20	5.11	5.68	5.63	6.57	14.64	Spo12905	NBS-LRR disease resistance protein	6.88 Kb downstream
S3_1196085	**G**:T	0.16	6.28	7.39	7.33	9.65	18.32	–	–	–
S3_1210582	C:**A**	0.20	9.00	8.31	3.91	12.13	27.30	Spo12821	CC-NBS-LRR disease resistance protein	2.07 Kb upstream
S3_1210599	**A**:G	0.20	5.28	5.62	5.78	7.30	15.19	Spo12821	CC-NBS-LRR disease resistance protein	2.06 Kb upstream
S3_1220905	A:**G**	0.20	7.46	7.26	4.02	10.81	23.22	Spo12821	CC-NBS-LRR disease resistance protein	0.98 Kb downstream
S3_1220957	T:**C**	0.20	7.46	7.26	4.02	10.81	23.22	Spo12821	CC-NBS-LRR disease resistance protein	1.03 Kb downstream
S3_1227676	T:**C**	0.18	6.55	6.11	3.53	9.43	19.48	Spo12821	CC-NBS-LRR disease resistance protein	7.75 Kb downstream
S3_1257152	A:**C**	0.19	5.74	6.23	5.07	9.50	16.88	–	–	–
S3_1257409	A:**G**	0.18	7.03	7.01	3.93	9.33	21.15	–	–	–
S3_1269788	T:**A**	0.20	7.07	7.89	4.00	10.41	21.66	–	–	–
S3_1269804	T:**C**	0.20	7.07	7.89	4.00	10.41	21.66	–	–	–
S3_1269906	A:**C**	0.18	5.68	5.69	3.60	9.46	16.79	–	–	–
Association panel comprises seedlings segregating only from a cross of Swan and Whale										
S3_325976	T:**C**	0.49	6.54	3.37	3.36	3.65	0.34	–	–	–
S3_325991	A:**C**	0.49	6.54	3.37	3.36	3.65	0.34	–	–	–
S3_326104	C:**T**	0.48	5.29	3.69	3.43	5.11	0.34	–	–	–
S3_692697^f^	A:**T**	0.46	6.68	3.68	3.44	4.22	0.35	Spo12784	NB-ARC; leucine-rich repeat (LRR)	2.69 Kb downstream
S3_948017^f^	C:**T**	0.17	5.62	3.45	–	5.11	0.34	Spo12903	Leucine-rich repeat (LRR); NB-ARC	18.00 Kb upstream
S3_954794^f^	T:**A**	0.17	5.62	3.45	–	5.11	0.34	Spo12903	Leucine-rich repeat (LRR); NB-ARC	11.23 Kb upstream
S3_1196085	**G**:T	0.19	5.32	3.51	2.89	2.39	0.32	–	–	–
S3_1210582	C:**A**	0.18	5.39	3.37	–	4.87	0.33	–	–	–
S3_1257152	A:**C**	0.19	5.25	3.47	–	4.73	0.32	–	–	–
S3_1257409	A:**G**	0.19	5.19	3.12	–	4.41	0.33	–	–	–

*^*a*^Genomic position of SNP marker on respective chromosomes. S3_325976 denotes the SNP marker on chromosome 3 positioned at 325976 bp. ^*b*^Beneficial alleles in bold are SNP alleles with positive effects and contributing to providing disease resistance. ^*c*^Association analysis was performed on four different programs. The principal components (PC) and kinship covariates were used in the TASSEL mixed linear model. Association analysis was conducted in GAPIT using the enriched compressed mixed linear model (EcMLM). The PC computed and used to run the logistic regression model in PLINK, and the genomic control (GC) association statistic was reported. Finally, a mixed model analysis in GENESIS was run using inbuilt PC-AiR and kinship matrices. ^*d*^Phenotypic variance (%) explained by the marker from the TASSEL mixed linear model. ^*e*^Candidate genes within the associated region were searched in the SpinachBase database (Collins et al., 2019; http://spinachbase.org/). The annotation and distance of the associated SNP from the candidate gene are reported in the table. ^*f*^These are uniquely associated SNPs only from the association panel comprising progeny population of Swan × Whale.*

**TABLE 3:** Haplotype-based association analysis at the race 13 of *P. effusa* resistance loci in spinach association panel comprising breeding populations from a cross of race 13 resistant cultivars Swan, squirrel, Tonga, T-Bird with susceptible cultivars Whale and Polka.

Haplotypes^a^			Allele frequency^b^		−log_10_ *P*-value^c^	
SNPs^d^	Allele^e^	Frequency^f^	Susceptible	Resistant	Logistic model	Linear model
**S3_325976**, **S3_325991**	CC	0.385	0.218	0.545	6.66	9.45
**S3_396000**, **S3_396030**	AA	0.454	0.253	0.648	8.84	12.78
**S3_948017**, **S3_954794**	TA	0.072	0.006	0.135	2.75	5.49
S3_1209978, S3_1209990	CG	0.140	0.018	0.259	4.52	9.83
**S3_1220905**, **S3_1220957**	GC	0.189	0.006	0.373	4.72	17.04
S3_1227442, S3_1227482, **S3_1227676**	TGC	0.169	0.007	0.324	5.19	14.82
**S3_1269788**, **S3_1269804**	AC	0.188	0.006	0.371	4.75	17.11

*^a^Haplotype blocks were identified using SNPs in the 0.3–1.3 Mb region of chromosome 3 in the Haploview program (Barrett et al., 2005) and the Plink v 1.70 (Purcell et al., 2007). ^b^Frequency of haplotype alleles among the resistant and susceptible groups. ^c^Haplotype association analysis was performed using logistic and linear regression models as implemented in Plink v1.70 (Purcell et al., 2007). ^d^The SNPs are named for chromosome and position. S3_325976 means SNP loci are located on chromosome 3 at 325976 bp. The SNPs in bold were also associated with a single marker association test, as listed in Table 2. ^e^Resistant haplotypes alleles in the population. Only beneficial haplotypes associated with the resistance were presented. ^f^Frequency of haplotype alleles in the population under study.*

A logistic regression model was run in PLINK using the PCA covariates computed in using the LD pruned SNPs and the IBS values calculated from the LD pruned SNPs. Nineteen SNP loci exceed the Bonferroni threshold in the PLINK logistic regression model (Table 2 and Figure 5C), of which ten SNPs associated in TASSEL MLM.

Finally, the logistic mixed model analysis was performed in the GENESIS R package. The genetic relatedness matrix estimated via PC-AiR and PC-Relate methods was used to fit a logistic model, and the Score test was used to assign the significance. Thirty loci were significantly associated with the race 13 resistance in the GENESIS model (Table 2 and Figure 5D). All the SNP markers detected by the TASSEL MLM and PLINK model were detected when analyzed in the GENESIS model.

To confirm genomic regions associated with resistance to race 13 of *P. effusa* from the association panels comprising multiple segregating populations, the second set of association analyses was conducted on progeny populations generated only from a cross of Swan and Whale. TASSEL mixed linear model identified 12 SNP loci (LOD value >5.2) significantly associated with the race 13 resistance from cultivar Swan, and all other tested association models showed higher LOD values for these SNPs (Table 2 and Supplementary Figure 2). Association analysis from this dataset uniquely identified three SNPs (S3_692697, S3_948017, and S3_954794) significantly associated with the race 13 resistance that was not detected from the whole panel.

### Haplotype Analysis in the Associated Region

**FIGURE 6:**
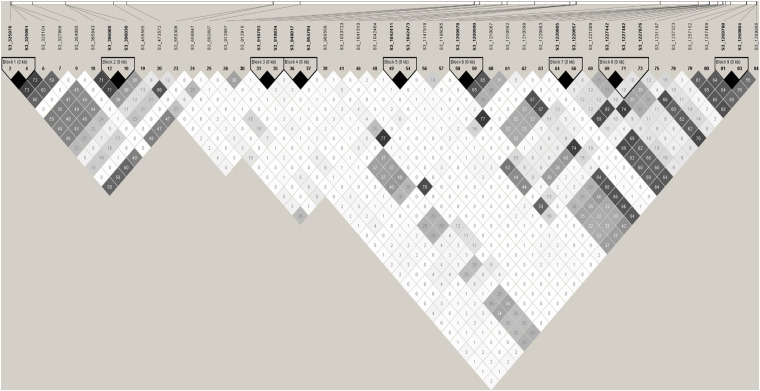
Haploview plot of pairwise linkage disequilibrium (LD) between SNPs associated with race 13 of *P. effusa* resistance region (0.30–1.30 Mb). LD is measured as *r*^2^ with maximum LD of colored as black and the minimum LD in shades of gray to white. Potential candidate genes close to the associated SNPs are listed in Table 2.

Haplotype analysis of the SNPs in the 0.30–1.30 Mb region of chromosome 3 in the whole association panel identified nine haplotype blocks (Figure 6). One haplotype blocks contain three SNPs, while all other contains two SNPs. Association analysis performed using the haplotypes as an independent marker identified seven haplotype bocks significantly associated with the race 13 resistance (Table 3), and the haplotype alleles associated with the downy mildew disease resistance were presented. On top of it, the 11 SNP markers associated with the single marker-based association models fell in the six haplotype blocks, and the multilocus SNP haplotypes from all six haplotype blocks were associated with the race 13 resistance (Figure 6). Significantly associated haplotypes were compared with the single SNP association result and were searched for potential disease resistance annotated genes in the reference genome.

Haploview plot (Figure 6) shows haplotypes at 0.32 and 0.39 Mb are in LD (*r*^2^ = 0.48) and in turn these regions are in LD with the SNP marker S3_692697 (*r*^2^ = 0.48-0.60). The SNP marker S3_692697 is not in LD with SNPs in 0.9–1.3 Mb. Similarly, the haplotypes S3_948017 and S3_954794 are in LD with the resistance associated SNPs: S3_1209978 (*r*^2^ = 0.37), S3_1220905 (*r*^2^ = 0.30), S3_1227676 (*r*^2^ = 0.25), S3_1269788 (*r*^2^ = 0.26). And the 1.22 Mb region is in high LD (*r*^2^ > 0.6) with 1.25–1.26 Mb region.

### Candidate Gene Analysis

**FIGURE 7:**
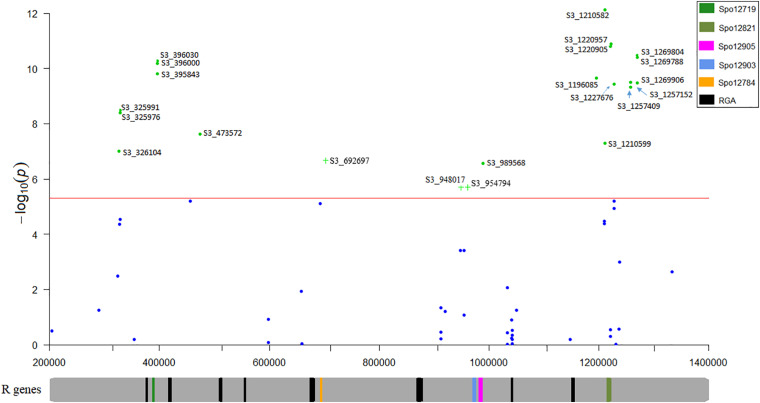
Regional association plot of the race 13 of *P. effusa* resistance in spinach. The horizontal and vertical axis represents the genomic position of the SNP and association power for each SNP with the trait expressed as –log_10_(*P*-value). The red line shows the Bonferroni-corrected genome wide threshold. The SNPs are named for chromosome and position. S3_325976 means SNP loci are located on chromosome 3 and positioned at 325976 bp. Plus (+) signs indicate SNP associations obtained from the second association panel composed of lines generated from a cross of Swan and Whale. Chromosomal location of the disease resistance candidate genes predicted to associate from this study, and other resistance genes between 0.30 and 1.30 Mb of spinach chromosome 3 were mapped.

Nineteen high confident SNPs that were identified in two or more association models were selected from the list of significant SNPs identified from multiple single marker association models. The race 13 resistance region was mapped between 0.32 and 1.26 Mb spanning 0.94 Mb of chromosome 3 using the mixed breeding population of 174 lines. A second association analysis performed on a subset of the original population (progeny population segregating from a cross of Swan and Whale) mapped the race 13 resistance loci explicitly to 0.69 and 0.94–0.95 Mb of chromosome 3. Genomic positions of the high-confidence SNPs consistently detected in tested association models were searched in the spinach reference genome to identify genes involved in disease resistance. All significant SNPs were laid on four physical regions (0.32–0.47, 0.69, 0.94–0.98, and 1.19–1.26 Mb) of the spinach chromosome 3 (Figure 7) that harbors disease resistance candidate genes: Spo12719, Spo12905, Spo12821, Spo12784, and Spo12903 (Table 2) within 0.98–18 Kb of the peak SNPs. Nine associated markers in the single marker-based association test formed a haplotype block in the Haploview analysis, and all the haplotype alleles were significantly associated with race 13 resistance endorsing association results obtained from the single marker test. Haplotypes block S3_396000- S3_396030, S3_948017-S3_954794, and S3_1220905-S3_1220957 showed a significant association with race 13 resistance and these blocks were 6 Kb of the gene Spo12719, 11 kb of the gene Spo12903, and 1 Kb of the gene Spo12821, respectively (Table 3 and Figures 6, 7). The 0.32 and 1.25–1.26 Mb regions associated with resistance to race 13 of *P. effusa* in this mapping effort did not contain any gene annotated to have functions related to disease resistance, which makes the candidate gene region to lie within from 0.39 to 1.22 Mb. In fact, both 0.32 and 1.25–1.26 Mb regions were in an LD (*r*^2^ > 0.50) to nearby regions (0.39 and 1.22 Mb) that contain disease resistance candidate genes (Figure 7). The SNPs in the 1.20–1.26 Mb regions are in LD, and these regions are in LD with the haplotype blocks in 0.94–0.95 Mb. Similarly, the 0.32 and 0.39 Mb regions are in LD with 0.69 Mb regions. Hence, the 0.69 and 0.94–0.95 Mb regions are likely associated with true functional *race* 13 resistance variants.

The five candidate genes (Spo12719, Spo12905, Spo12821, Spo12784, and Spo12903) lying within or near the associated SNP or LD blocks were annotated as a receptor-like kinase, NBS-LRR disease resistance protein, NB-ARC-LRR disease resistance protein, and CC-NBS-LRR disease resistance protein in the SpinachBase^1^ (Collins et al., 2019). All the identified candidate gene families are involved with functions in the plant disease resistance mechanism. The proximal end of chromosome 3 contains several other annotated disease resistance genes (Xu et al., 2017; She et al., 2018) and the markers for the *RPF*1, *RPF*2, and *RPF*3 (Irish et al., 2008; Feng et al., 2018a) fall in the same region.

## Discussion

### Downy Mildew Disease Resistance

Downy mildew is the economically most important disease of spinach affecting commercial spinach production worldwide and in the United States and Europe. Downy mildew infected leaves are unmarketable and involve an extra cost in manual culling of the infected plants in commercial production (Feng et al., 2018b). Genetic resistance offers an efficient disease control method and many resistant spinach cultivars have been released (Correll et al., 2011). However, the emergence of new races of *P. effusa* that are overcoming the genetic resistance deployed in the new cultivars (Correll et al., 2011; Feng et al., 2018a) is the major challenge in breeding for downy mildew resistance. Therefore, a deeper understanding of host–pathogen interaction that mainly includes identifying and mapping multiple resistance sources, a functional test of the *RPF* genes, and characterizing functions of the effector genes are prioritized. Mechanistic understanding of spinach-downy mildew interaction, characterization of virulence evolution of the downy mildew races, and functional characterization of genetic resistance may contribute toward formulating strategic implementation of the genetic resistances to manage downy mildew disease.

Undoubtedly, there is an urgent need for stable resistant cultivars against all known downy mildew races. Identification of tightly linked markers for each *RPF* locus against each races of *P. effusa* will enhance the efficiency and precision of developing downy mildew resistance cultivars. The DNA markers tagged to the downy mildew resistance loci are being developed in spinach (Irish et al., 2008; Feng et al., 2015, 2018a), and the development of functional gene-based markers will ease the pyramiding or stacking of several resistance sources (multiple *RPF* loci) into a single cultivar. Cultivars with multiple resistant genes may be durably resistant and are attractive options for the spinach industry as the evolution of new races against multiple R genes is less likely to occur (McDonald and Linde, 2002).

Multiple spinach segregating populations were phenotyped for resistance against race 13 of *P. effusa*. The F2 populations generated from a cross between heterozygous resistant and susceptible parents segregated with the expected segregation of 1:1 resistant to susceptible; however, several populations did not segregate 1:1 (Table 1). Some possible explanations could be the plants in the crossing blocks were a mixture of heterozygous F1 (Rr) and homozygous recessives (rr) as the F1 parents were not tested with *P. effusa* or the *RPF* markers. The F1 seeds were produced by crossing two inbred lines, one of which has the *RPF*1 locus to generate the resistant F1 hybrid cultivar, although crosses among the plants of the same cultivar might have occurred in some of the isolators generating some inbreds in the seed lots. Spinach is a dioecious crop, with separate male and female plants, although, some monoecious plants are found (Morelock and Correll, 2008). It is often difficult to generate an inbred line, and the parent lines used in the crosses are family pools of heterozygous genotypes making the linkage and QTL analysis more difficult in spinach. However, association analysis allows us to map the trait in a mixed population or a panel of diverse germplasm accessions. Thus, populations generated via by crossing a resistant and susceptible cultivar were genotyped and phenotyped for multiple populations and were pooled to conduct an association analysis to map the race 13 resistance loci. We seek to identify significantly associated SNP markers, to extend their use as a Kompetitive allele specific PCR (KASP) (Semagn et al., 2014) and high resolution melting (HRM) (Simko, 2016) diagnostic marker assays, and narrow down the race 13 resistance locus. High throughput genotyping methods as KASP and HRM are more desirable compared to laborious gel-based marker assays and have been applied in several crops (Baumgartner et al., 2016; Noh et al., 2018; Salinas et al., 2020).

### GWAS Analysis for Downy Mildew Resistance

Spinach population segregating from four cultivars were evaluated for resistance against the race 13 of the downy mildew. The GWAS population was derived by crossing genetically unique cultivars to generate the F1 and subsequent F2 generation. The resulting familial population allowed exploiting LD as it would extend to longer genetic intervals, unlike short LD decay in the population consisting of diverse natural germplasm accessions. As such, with larger LD blocks passing on to the progeny population, association analysis within a family is expected to provide increased power and resolution in identifying SNP association and candidate genes. The spinach association panel was subdivided into four sub-populations in the PCA and the ADMIXTURE analysis. The PCA and kinship matrices were used as a covariate in the mixed model analysis in TASSEL, GAPIT, PLINK, and GENESIS models. Association results were reported following the Bonferroni correction threshold to control false-positive associations.

Association models were run in multiple programs to generate consensus sets of significant SNPs. Association analysis in this study authenticates a narrow region governing the genetic basis of resistance to race 13 of *P. effusa* on chromosome 3. The MLM model in TASSEL that accounts for both population structure and kinship identified very similar sets of SNPs as with the EcMLM model in GAPIT (Table 2). In both models, the SNPs at 0.32 Mb of chromosome 3 were not significant. The PLINK model identified the least number of associated SNPs and did not show substantial associations to SNPs at 1.22 Mb and onward. The GENESIS model captured the SNPs that were independently identified by all the other three models. Alternatively, association analysis conducted on a set of lines segregating from Swan and Whale showed strong associations in a 0.69 and 0.94–0.95 Mb regions, and these regions were uniquely identified only from this population.

Haplotype analysis formed nine haplotype blocks in the 0.3–1.3 Mb region of chromosome 3. Six haplotype blocks were significantly associated with resistance to race 13 of *P. effusa*. A haplotype block size ranged from 0.1–6 Kb, and most of the haplotypes blocks were formed with two SNPs. Haplotype analysis can be useful in identifying marker-trait association and can often identify unique haplotype alleles associated with the trait that might not associate in a single SNP marker test. Haplotype association analysis in this study identified SNP haplotypes associated with the resistance that was also identified via a single SNP marker association test that corroborate the association report from single SNP marker analysis. Association result from this study falls in the same region as the previously reported *RPF* locus in spinach (Feng et al., 2018a; She et al., 2018), and this report thus illustrates efficient use of a small panel of multiparent progenies to conduct association analysis toward identifying and mapping the genes. However, multiple haplotype regions were in high LD and such non-random associations between SNPs in the gene dense areas limit the resolution in identifying the true causal variants and associated genes.

Downy mildew resistance in spinach is hypothesized to be governed mainly by a major genes with a substantial effect on phenotype. Despite expected high LOD value and *R*^2^ values for the resistant locus, a medium LOD and *R*^2^ values were observed for the SNP markers associated with the race 13 resistance. The medium- LOD value might be because that the associated SNPs are still far apart from the functional allele and the candidate genes. Spinach being an open-pollinated heterozygous species, linkage disequilibrium decay is faster, which is estimated at around 4 Kb (Xu et al., 2017). On the other hand, multiple moderate effect genes or a gene with multiple alleles might control the resistance, and so each SNP regions showed relatively lower LOD values. Despite the moderate LOD and *R*^2^ values, the current result provided a high-resolution characterization of the race 13 resistance locus. These results plus a further understanding of the genetic mechanism underlying the downy mildew resistance mechanism may be helpful in the efficient and effective deployment of the resistance alleles.

### Downy Mildew Resistance Candidate Genes

Association analysis was performed in this study to map the resistance locus against race 13 of *P. effusa* using segregating populations from resistant cultivars Swan, Squirrel, Tonga, and T-Bird. The resistance region was mapped to chromosome 3, and the associated SNPs lie in 0.39, 0.47, 0.69, 0.94–0.98, 1.19, and 1.21–1.22 Mb of chromosome 3. Indeed, many of the single marker SNP associated with downy mildew resistance also formed the LD block, and their haplotype alleles were associated with the *P. effusa* resistance, providing increased confidence of this mapping result.

The spinach chromosome 3 (0.34–1.26) contains 14 genes annotated to have plant disease resistance properties (Xu et al., 2017; She et al., 2018; Collins et al., 2019). The DM1 marker tightly linked to the *RPF*1 locus (Irish et al., 2008), and *RPF*1, *RPF*2, and *RPF*3 loci were mapped in the same region (Feng et al., 2018a). The *RPF*1 candidate gene region was narrowed to 1.5 Mb region in Feng et al. (2018a) and 0.89 Mb region in (She et al., 2018). Five genes (Spo12736, Spo12784, Spo12903, Spo12905, and Spo12821) close to the DM1 marker in the Sp75 genome were predicted as downy mildew resistance candidate genes (Xu et al., 2017) while the study of She et al. (2018) reported Spo12729, Spo12784, and Spo12903 as the most likely candidate genes. However, the spinach genome sequence of inbred line Sp75 does not contain the *RPF*1 locus but may contain other disease resistance genes, and the candidate genes postulated based on the Sp75 gene models may not be specific for the *RPF*1 locus.

Significant SNPs identified in this study do not fall in the gene regions annotated to have plant disease resistance function. However, most of the significantly associated SNPs were within 1–7 Kb of the plant defense genes Spo12719, Spo12905, Spo12821, and Spo12784 annotated as a receptor-like kinase, NBS-LRR disease resistance protein, CC-NBS-LRR disease resistance protein, and NB-ARC LRR proteins. Only the Spo12903 gene annotated as NB-ARC LRR protein is 11–18 Kb from the significant SNPs. The LRR-NBS domains are the most common plant disease resistance gene that acts as a receptor of pathogen effectors to activate the signaling cascades for defense (Jones and Dangl, 2006). The region associated with resistance to race 13 of *P. effusa* in this study falls in the putative downy mildew resistance gene region that contains 14 disease resistance genes in 0.90 Mb. Genes Spo12784, Spo12903, Spo12905, and Spo12821 identified as potential candidate genes in this study are the closest genes to the significantly associated SNPs, and these genes were postulated as candidate downy mildew resistance genes in spinach (Xu et al., 2017). In contrast, only Spo12784 and Spo12903 genes identified in this study were postulated as the candidate genes following amino acid sequence analysis and conserved domain analysis in spinach inbreed lines (She et al., 2018). The candidate genes identified from the spinach genome of Xu et al. (2017) may not be specific to the *RPF*1 locus. However, the annotated genes predicted to provide plant disease resistance in the spinach reference genome might be members of the gene cluster providing resistance against spinach downy mildew pathogen. Overall, a prioritized list of candidate genes associated with resistance functions was identified in this study based on association analysis results from a single marker test, multimarker haplotype test, and LD pattern in the associated regions.

The spinach downy mildew resistant locus *RPF*1-6 has been established and is being characterized at the genetic and functional level, although much effort and emphasis have been focused on cloning of the *RPF*1 gene. Coordinated efforts in discovering and describing the major and minor downy mildew resistance genes are pursued to combat the rapidly evolving new virulent races to minimize the effect of resistance breakdown. Detailed genetic characterization of the resistance genes allows molecular breeding with an increased selection efficiency in terms of time and precision to deploy the resistant alleles during cultivar development. On the other hand, functional characterization of the R genes will allow understanding of the genetic and functional mechanism of host–pathogen interaction and disease development, mechanism of evolution of the new virulent races, and the pathogen strategy to overcome the available resistances. Such information will be useful to formulate a new strategy in spinach breeding and cultivar development and to provide an explained model of the host–pathogen interaction to translate in crop disease management and breeding.

The development of functional markers residing on the candidate disease resistance gene is most desirable. Still, it warrants gene identification and cloning with explained functions of the domains toward resistance-susceptibility. Development of genetically linked and associated markers are commonly used in plant breeding programs to select plants with expected phenotype from the genotype data. Multiple resistance genes can be introduced and stacked using the DNA markers as reported for three bacterial blight resistance genes in rice (Pradhan et al., 2015). Such multiple gene stacking is considered to be more durable as the new races are rapidly breaking down the single R genes. Furthermore, identification of functional genes involved in the downy mildew resistance mechanism opens spectrum to stack multiple R genes cassettes that can be introduced as a single locus (Wulff et al., 2011; Zhu et al., 2012). In addition, continual advancements in sequencing platforms and reduction of sequencing cost, it is now possible to sequence a panel of plant genome or transcriptome at a reasonable price. Whole-genome resequencing of spinach core collections is ongoing, and the sequence-based genomic resources and millions of SNP of the core collections will be available soon. The new genomic resources will allow and facilitate an expanded understanding of the biology of commercially important traits, including the gene variation in the downy mildew resistance locus. As an alternative to resistance gene-based disease control, there is an increasing interest in exploring the susceptibility genes as in barley against powdery mildew (Büschges et al., 1997) and downy mildew in *Arabidopsis thaliana* (Van Damme et al., 2008, 2009). Knockout or silenced inactivation of the susceptibility genes was demonstrated to provide disease resistance against many pathogens, including rice blast (Wang et al., 2016), powdery mildew in grapes (Pessina et al., 2016), and tomatoes (Bai et al., 2008). Identifying the *S* genes in spinach and their use might help to minimize production loss and transient effectiveness of the known major *RPF* genes because of the rapid evolution potential of the downy mildew pathogens.

We performed a GWAS analysis in a set of 174 spinach lines segregating for resistance from four cultivars, and a subset of 65 lines segregating from two cultivars Swan and Whale. We identified some major loci resistant to race 13 of *P. effusa*. The SNP loci are close to the genes annotated to govern disease resistance, and the favorable allele can be used in spinach breeding to select for the resistance genotypes through marker-assisted selection approaches. Validation of candidate genes Spo12784 and Spo12903 as priority and Spo12905 and Spo12821 in second priority via gene-knockout and gene-expression experiments should be explored to uncover the downy mildew resistance mechanism and control. Current research efforts aim to expand the understanding of host–pathogen interaction in spinach downy mildew. The main focus is in identifying and mapping multiple resistance sources, performing a functional test of the *RPF* genes and characterizing functions of the effector genes, to gain insights toward identifying susceptibility genes and factors. Rapidly emerging races are breaking down the resistance deployed in commercial cultivars, and the host–pathogen battle in spinach downy mildew system offers a model to understand and explore the continued host–pathogen win-lose interactions. All new understanding of resistance-susceptibility genetics may help in formulating and adopting an improved downy mildew resistance breeding strategy. Identification of prioritized candidate gene list and future reports on an expanded understanding of spinach-downy mildew host–pathogen interaction and functional characterization of genetic resistance-susceptibility will be of high value in the scientific community and in adopting an improved strategy to use the genetic resistance against the downy mildew.

## Conclusion

The current study identified downy mildew resistance loci using a population segregating from cultivars Swan, Squirrel, Tonga, and T-Bird. We used the association mapping in the breeding population to map the resistant loci. SNPs in 0.39, 0.69, 0.94–0.98, and 1.2 Mb of chromosome 3 were significantly associated with resistance to race 13 of *P. effusa* in spinach panel. The SNPs were located within 1–7 kb of the disease resistance genes Spo12784, Spo12719, Spo12905, and Spo12821, and 11–18 Kb from Spo12903. Sp012784 and Spo12903 are most likely candidate genes providing downy mildew resistance in spinach, and these two genes should be targeted for functional validation tests. Candidate genomic regions associated with the race 13 of *P. effusa* resistance and continual development of race-specific resistance will enhance efficiency and precision of developing downy mildew resistant cultivars. Indeed identification of new resistance loci and linked DNA markers will make the pyramiding or stacking of several resistance sources (multiple *RPF* loci) into a single cultivar feasible. A commercial cultivar with multiple resistant genes is an attractive option in the spinach industry as the cultivar will be durably resistant owing to resistances of spinach lines against several races of *P. effusa*.

## Data Availability Statement

The datasets generated for this study can be found in FigShare, https://doi.org/10.6084/m9.figshare.12870998.v1.

## Author Contributions

GB, AS, JC, and BM conceived the study. BM conducted crossing. GB planned and performed the phenotyping, genotyping, analysis, and drafting of the manuscript. GB, AS, CF, and BD revised the manuscript. AS, JC, and BM reviewed, revised, and approved the final version of the manuscript. All authors contributed to the article and approved the submitted version.

## Conflict of Interest

The authors declare that the research was conducted in the absence of any commercial or financial relationships that could be construed as a potential conflict of interest.

## Footnotes

1 www.spinachbase.org

## Supplementary Material

Supplementary Figure 1 Population structure of the spinach lines generated from a cross of cultivars Swan and Whale. **(A)** Optimum K was determined using the minimum cross-validation errors in the data for K. **(B)** Classification of spinach genotypes in the association panel into two genetic sub-populations. The horizontal axis represents the spinach genotypes, and the vertical axis of the plot represents the probability of genotypes belonging to different genetic groups. Two colors indicate membership proportion to each population group.

Supplementary Figure 2 Manhattan and QQ-plots of genome wide associations of the race 13 of *P. effusa* resistance in spinach population generated from a cross of Swan and Whale in TASSEL **(A)**, GAPIT **(B)**, PLINK **(C)**, and GENESIS **(D)** model. The horizontal and vertical axis represents the genomic position of the SNP and association power for each SNP with the trait expressed as −log_10_ (*P*-value). The dashed line shows the Bonferroni-corrected genome wide threshold.
