# Supplementary figures and images for: Genome Wide Association Studies in Multiple Spinach Breeding Populations Refine Downy Mildew Race 13 Resistance Genes
Source: Front Plant Sci. 2020 Oct 21;11:563187. doi: 10.3389/fpls.2020.563187 (PMC7609621; doi:10.3389/fpls.2020.563187)

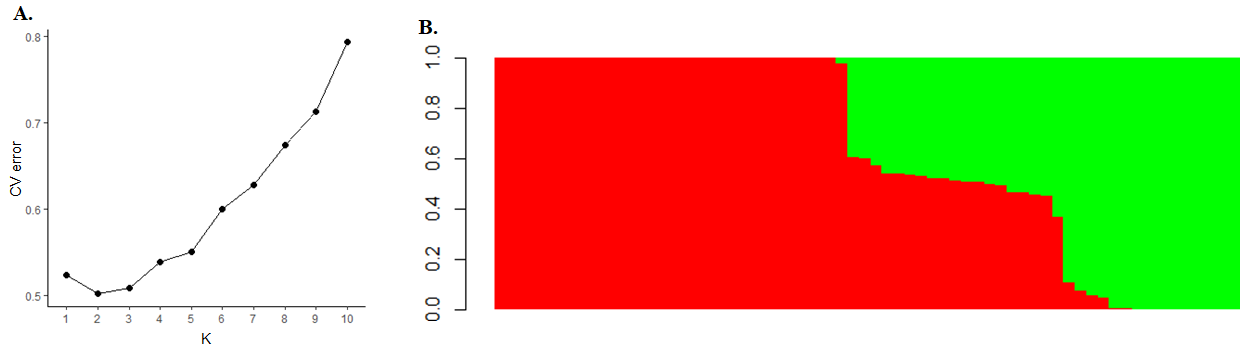

Supplement: Supplementary Figure 1 — Population structure of the spinach lines generated from a cross of cultivars Swan and Whale. (A) Optimum K was determined using the minimum cross-validation errors in the data for K. (B) Classification of spinach genotypes in the association panel into two genetic sub-populations. The horizontal axis represents the spinach genotypes, and the vertical axis of the plot represents the probability of genotypes belonging to different genetic groups. Two colors indicate membership proportion to each population group. [file Image_1.TIF]

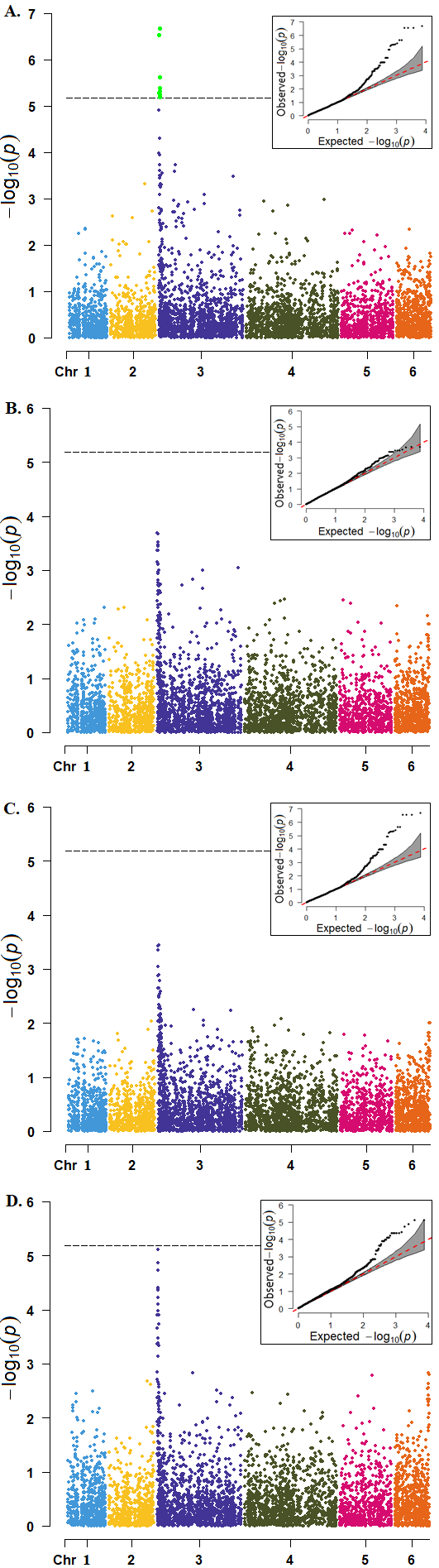

Supplement: Supplementary Figure 2 — Manhattan and QQ-plots of genome wide associations of the race 13 of P. effusa resistance in spinach population generated from a cross of Swan and Whale in TASSEL (A), GAPIT (B), PLINK (C), and GENESIS (D) model. The horizontal and vertical axis represents the genomic position of the SNP and association power for each SNP with the trait expressed as −log10 (P-value). The dashed line shows the Bonferroni-corrected genome wide threshold. [file Image_2.TIF]
